# Spatial Scales of Genetic Structure in Free-Standing and Strangler Figs (*Ficus*, Moraceae) Inhabiting Neotropical Forests

**DOI:** 10.1371/journal.pone.0133581

**Published:** 2015-07-30

**Authors:** Katrin Heer, Elisabeth K. V. Kalko, Larissa Albrecht, Roosevelt García-Villacorta, Felix C. Staeps, Edward Allen Herre, Christopher W. Dick

**Affiliations:** 1 Conservation Biology, Faculty of Biology, Philipps-University Marburg, Karl-von-Frisch-Strasse 8, 35043 Marburg, Germany; 2 Department of Ecology, Faculty of Biology, Philipps-University Marburg, Karl-von-Frisch-Strasse 8, 35043 Marburg, Germany; 3 Institute of Experimental Ecology, University of Ulm, Albert-Einstein-Allee 11, 89069 Ulm, Germany; 4 Smithsonian Tropical Research Institute, P.O. Box 0843–03092, Balboa, Panamá; 5 Institute of Molecular Plant Sciences, University of Edinburgh, Mayfield Rd, Edinburgh EH9 3JH, United Kingdom; 6 Department of Ecology and Evolutionary Biology, University of Michigan, 830 North University, 2019 Kraus Natural Science Building, Ann Arbor, MI 48109–1048 United States of America; University of Innsbruck, AUSTRIA

## Abstract

Wind-borne pollinating wasps (Agaonidae) can transport fig (*Ficus *sp., Moraceae) pollen over enormous distances (> 100 km). Because of their extensive breeding areas, Neotropical figs are expected to exhibit weak patterns of genetic structure at local and regional scales. We evaluated genetic structure at the regional to continental scale (Panama, Costa Rica, and Peru) for the free-standing fig species *Ficus insipida*. Genetic differentiation was detected only at distances > 300 km (Jost´s *D_est_* = 0.68 ± 0.07 & *F_ST_* = 0.30 ± 0.03 between Mesoamerican and Amazonian sites) and evidence for phylogeographic structure (*R*_ST_>>permuted *R*_ST_) was only significant in comparisons between Central and South America. Further, we assessed local scale spatial genetic structure (SGS, d ≤ 8 km) in Panama and developed an agent-based model parameterized with data from *F*. *insipida* to estimate minimum pollination distances, which determine the contribution of pollen dispersal on SGS. The local scale data for *F*. *insipida* was compared to SGS data collected for an additional free-standing fig, *F*. *yoponensis* (subgenus *Pharmacosycea*), and two species of strangler figs, *F*. *citrifolia* and *F*. *obtusifolia* (subgenus *Urostigma*) sampled in Panama. All four species displayed significant SGS (mean *Sp* = 0.014 ± 0.012). Model simulations indicated that most pollination events likely occur at distances > > 1 km, largely ruling out spatially limited pollen dispersal as the determinant of SGS in *F*. *insipida* and, by extension, the other fig species. Our results are consistent with the view that *Ficus* develops fine-scale SGS primarily as a result of localized seed dispersal and/or clumped seedling establishment despite extensive long-distance pollen dispersal. We discuss several ecological and life history factors that could have species- or subgenus-specific impacts on the genetic structure of Neotropical figs.

## Introduction

In contrast with high latitude forests, where wind is often the primary vector of pollen- and seed-mediated gene flow in trees, animals are the predominant pollinators and dispersers of tropical trees [1,2]. Biotic interactions thus play a vital role in gene flow and the maintenance of genetic diversity in tropical trees, and genetic based parentage analyses have shown that insects frequently transport pollen over hundreds to thousands of meters (reviewed in [2]). To date, the greatest insect-mediated pollen dispersal distance of 160 km [3] has been recorded for a fig tree (*Ficus* spp., Moraceae). Fig trees have specialized, often species-specific relationships with minute (1–2 mm) wasps in the family Agaonidae [4,5,6]). Although fig-pollinating wasps are weak flyers and live only 1–2 days, they can be carried long distances by wind currents [3,7]. Figs, particularly in the Neotropics, typically occur at low population densities (e.g. one adult/ha). Further, they flower asynchronously, which increases the already large distances between potential mates [4]. Thus, large pollen dispersal distances in figs are the rule rather than the exception and breeding areas are considered to be huge [7]. Some authors have even suggested that figs might be panmictic at regional spatial scales [8,9].

There is some debate about how patterns of gene dispersal distances, and therefore spatial genetic structure (SGS), are likely to differ among *Ficus* life forms and subgenera [10,11,12]. For example, functionally dioecious figs of Paleotropical forests are often small in stature, and have clumped distributions and locally dispersing wasp pollinators [10,11,13], traits which could impede long distance gene flow and enhance SGS relative to monoecious species (Table 1). Dev et al. [11] hypothesized that Neotropical species should exhibit weaker SGS than the Indian species in their study, and in dioecious Paleotropical *Ficus* more generally. Nazareno et al. [12] subsequently performed an SGS analysis of two fig species (subgenus *Urostigma*) in Brazil and indeed found evidence of significantly lower SGS as measured by the *Sp* statistic, which measures degree of SGS [14].

**Table 1:** Characteristics of the four studied Neotropical *Ficus* species. Calculation of Nearest Neighbour distance (median with 25th /75th percentile in parentheses) is based on census data by Albrecht, Kalko and Handley.

Species	*Ficus insipida* Willd.	*Ficus yoponensis* Desv.	*Ficus citrifolia* Mill.	*Ficus obtusifolia* Kunth
Subgenus	*Pharmacosycea*		*Urostigma*	
Growth form	large, free-standing trees		mostly stranglers or hemi-epiphytes	
Regeneration	in light gaps		on host trees	
Flowering pattern	asynchronous in population			
Pollen dispersal distances	wide ranging (> 10 km)			
Main seed dispersers	frugivorous bats (Phyllostomidae)			
Pollinating fig wasp	*Tetrapus costaricanus* Grandi, 1925	*Tetrapus ecuadoranus* Grandi, 1934	*Pegoscapus tonduzi* Grandi, 1919	*Pegoscapus Hoffmeyeri* Grandi, 1934
Fruit weight	9.5 ± 1.3 g	3.1 ± 0.7 g	1.6 ± 0.3 g	9.5 ± 1.4 g
Tree density (in m)	36 (16–87)^1^	94 (37–208)^1^	98 (38–231)^1^	99 (49–209)^1^
References	[65,78]			

Within the Neotropical *Ficus*, the two subgenera exhibit contrasting life histories that could further influence genetic structure (see Table 1). For example, free-standing figs (subgenus *Pharmacosycea*) require high light intensities mostly encountered in large light gaps [15], which could lead to clumped pattern of seedling recruitment. In contrast, strangler figs (subgenus *Urostigma*) establish as hemi-epiphytes on host trees, rendering effective seed dispersal a more spatially random process.

At the biogeographic scale, genetic structure has been shown for several fig species [16,17]. A recent study of the free-standing Neotropical species *Ficus insipida* subs. *insipida* showed significant cpDNA haplotype differentiation between Mesoamerican and western Amazon samples [18]. Using a molecular clock approach, these authors suggested a mid-Miocene (14.6 Ma, range 5.1 to 26.4 Ma) divergence time of the South and Central American haplotypes. Given the absence of differentiation at the nuclear ITS locus, the authors suggested that the phylogeographic structure was caused primarily by seed dispersal limitation combined with the geographically isolating effects of the northern Andes and Llanos region.

Here we present an analysis of genetic structure in Neotropical figs based on nuclear microsatellite data. Our study spans local, regional, and continental spatial scales for *Ficus insipida*, and compares local scale spatial genetic structure (SGS) in an additional free-standing fig, *F*. *yoponensis* (subgenus *Pharmacosycea*), and two species of strangler figs, *F*. *citrifolia* and *F*. *obtusifolia* (subgenus *Urostigma*) within Panama. Using data from *F*. *insipida*, we developed and parameterized an agent-based model to estimate minimum pollen dispersal distances. We then evaluated the extent to which pollen dispersal is expected to drive SGS in our study species. Finally, in order to determine whether our microsatellite data concurs with results from plastid and ITS sequences in showing population differentiation for *F*. *insipida* across its distribution range [18], we sampled the widespread species *F*. *insipida* (subgenus *Pharmacosycea*) broadly within the Panama Canal Watershed, and in Costa Rica and Peru and tested for phylogeographic structure at these nuclear loci.

## Material & Methods

### Studied species

There are more than 750 *Ficus* species (family Moraceae) worldwide [19,20] from monoecious and functionally dioecious subgenera. In the Neotropics, only monoecious species of two of the four acknowledged fig subgenera are present [21]. Molecular and morphological evidence suggest that subgenus *Pharmacosycea* is phylogenetically distant from *Americana* [22]. Trees of the subgenus *Pharmacosycea* are pollinated by fig wasps of the genus *Tetrapus* (Agaonidae) and grow as free-standing trees that mostly establish in early successional stages due to their initial dependency on high light intensity [15]. Species belonging to the subgenus *Urostigma* (section *Americana*) grow mostly as hemi-epiphytes or strangler figs on host trees (hereafter we will refer to *Urostigma* as “strangler figs”). *Americana* figs are pollinated by fig wasps of the genus *Pegoscapus* [19].

In our study area in Panama, 13 of 16 fig species ripen medium to large, green, inconspicuous fruits that are mostly removed at night [23] suggesting that the seeds are mainly dispersed by fruit-eating bats of the Neotropical family of leaf-nosed bats (Phyllostomidae, Chiroptera). We concentrated our study on the most common and widespread species *F*. *insipida*. At the local scale we included a second free-standing fig species *F*. *yoponensis*, and two species of strangler figs, *F*. *citrifolia* and *F*. *obtusifolia*, all of which are mainly dispersed by fruit-eating bats. *Ficus obtusifolia* usually grows as a strangler fig or hemi-epiphyte, and seldom as free-standing tree. Large individuals are most frequently found in mature forests. In contrast, *F*. *citrifolia* mostly grows as a small hemi-epiphyte or bush and is frequently found on palm trees in the study area (see Table 1 for more details on the studied species).

### Study sites and sampling

**Fig 1:**
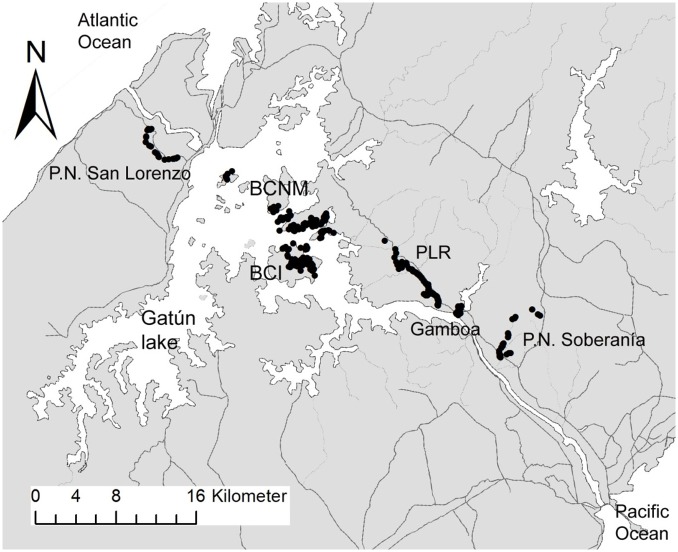
Sampling sites of *F*. *insipida* located along the Panama Canal. Black dots indicate sampled individuals. (BCNM: Barro Colorado Nature Monument, BCI: Barro Colorado Island, PLR: Pipeline Road, detailed maps for all species sampled in BCNM are presented in S3 Fig).

The study of local SGS was conducted in tropical moist forest on several islands and peninsulas within the Barro Colorado Nature Monument (BCNM, Fig 1 and S1 Fig), Republic of Panama. Annual rainfall at BCNM averages 2,600 mm and there is a pronounced dry season from mid-December to mid-April. The largest island in the BCNM with about 15.6 km^2^ is Barro Colorado Island (BCI) where adult figs have been censused repeatedly since 1973 [24,25]. Since 1982, fig populations have been censused along the shoreline of many additional islands and peninsulas (Albrecht, Herre, unpubl. data), and we collected samples of a high number of individuals despite the low population densities of the four targeted fig species. The free-standing *F*. *insipida* (n = 190) is by far the most common fig species in the vicinity of the Panama Canal and relatively abundant along the shoreline as well as on the North-Eastern part of BCI which is covered by secondary forest of about 90–150 years [26]. In contrast, the free-standing fig *F*. *yoponensis* (n = 37) is relatively rare along most shorelines and thus sampling was limited to the secondary forest on BCI. The strangler figs *F*. *citrifolia* (n = 62) and *F*. *obtusifolia* (n = 59) were mostly sampled along the shoreline, as few individuals are known from the core region of the main island (S1 Fig). Because exhaustive census data are restricted to the main island and to the shoreline of the surrounding islands and peninsulas, we were not able to determine the overall fig tree density but calculated Nearest Neighbor (NN) distances based on all censused individuals (Table 1).

Our focal species *F*. *insipida* was additionally sampled at several sites within the Panama Canal watershed. First, we collected samples along a 7 km stretch of Pipeline Road (PLR in the following, n = 155), within in Soberanía National Park, for more intensive studies of spatial genetic structure, including a comparison of SGS in adults (dbh ≥ 10 cm, n = 67) and saplings (< 3 m in height and with soft leaves, n = 73). While mostly situated within 20 m from the dirt road, some individuals were located up to 600 m in the forest interior (S1 Fig). At the regional scale, we sampled *F*. *insipida* in the eastern sector of Soberanía National Park, close to the town of Gamboa, and in Parque Nacional San Lorenzo on the Caribbean Coast. All sampling sites were located close to the Panama Canal and spanned a total distance of about 43 km (Fig 1). To test for genetic differentiation over biogeographic scales, we sampled *F*. *insipida* from Costa Rica and northern Amazonian Peru. In Costa Rica, samples were collected in and around Tirimbina Ecological Reserve in Sarapiqui. In Peru, samples were obtained from sites in the Peruvian regions Loreto and San Martín (see S1 Table for geographic coordinates and numbers of samples for each site).

Leaves were collected either directly from the crown or in a few cases from the ground below the canopy if there were no conspecific trees nearby and if the leaves were still green. All leaf samples were immediately transferred to Ziploc bags containing silica gel. Due to the generally low tree densities and the concentration of most individuals along the shorelines or forest roads, we did not follow a particular sampling scheme [14,27], but rather sampled as many individuals as feasible.

### Ethic statement

Permits for sample collection in protected areas and sample export were obtained from the Autoridad Nacional del Ambiente, Panama (ANAM, Permit No. SC/AP-2-10 and SEX/AP-8-10), Ministerio del Ambiente, Energía y Telecomunicaciones, Costa Rica (MINAET, Permit No. 047–142 2010_SINAC) and Instituto Nacional de Recursos Naturales, Peru (INRENA, Autorización No. 029 -2004-INRENA-IFFS-DCB).

### Genotyping

DNA was extracted from ca. 10 mg of desiccated leaf tissue using a modified CTAB protocol [28]. We scored 7 to 10 nuclear microsatellite loci for each species (S2 Table) with markers that were developed from an EST library of *F*. *citrifolia* and *F*. *popenoei* [29] or were published previously [30,31]. Details on PCR conditions and genotyping are described in Heer et al. [29]. We used one fluorescently labeled primer that could be applied universally with all microsatellites via an attached M13 adaptor sequence [32]. For genotyping, we pooled 2–4 loci using 1 μl of each PCR product in 12 μl formamide and a ROX500 internal standard (Applied Biosystems, Carlsbad, CA, USA). Genotyping was performed in an ABI Prism 3730 Sequencer. Alleles were scored with GeneMarker V1.97 Demo Version (SoftGenetics, State College, PA, USA). Samples were only included in the final analysis when ≥ 60% of the loci could be scored. We also tested a number of maternally inherited chloroplast markers (cpSSR) in order to obtain additional information on seed dispersal distances but none of them was polymorphic within the Panama Canal watershed (see S1 Methods for details). *Microsatellite data are within Supporting Information files* (S1 Dataset).

### Genetic Data Analysis

For each species and sampling site we calculated total numbers of alleles (*N*_A_), expected heterozygosity (*H*_*e*_), global estimates of *F*_*IS*_ over alleles [33] and tested for deviations from the Hardy-Weinberg equilibrium (HWE) using Genepop 4.1 [34]. Presence of null alleles was determined with MicroChecker 2.2.3 [35]. If null alleles were present, we calculated the mean null allele frequency from the four estimates provided by the software.

For the local scale SGS analyses, we calculated the kinship coefficient *F*_*ij*_ proposed by Loiselle et al. [36] that measures genetic similarity between individuals *i* and *j* relative to the mean similarity of any random pair of individuals in the sample. This statistic is robust even in the presence of alleles with low frequencies [14]. With the information on spatial and genetic distance between individuals we compared spatial genetic structure among cohorts, populations and species with the *Sp* statistic, which is a summary statistic introduced by Vekemans and Hardy [14]. This statistic largely circumvents the limitations of other methods (e.g. spatial autocorrelation) that are strongly dependent on sampling scheme and arbitrary choice of distance classes. The *Sp* statistic is defined as $Sp=\frac{-b}{\left(1-F_{1}\right)}$ with *b* as the slope of the regression of *F*_*ij*_ on ln(*d*_*ij*_), the natural logarithm of the spatial distance between individuals, and *F*_*1*_ being the mean pairwise kinship coefficient of the first distance class that includes nearest neighbors (with *d*_*ij*_ < 200 m for free-standing figs and *d*_*ij*_ < 300 m for strangler figs taking account of the density of sampled individuals). To determine the significance of *b* we conducted 9999 permutations of the spatial positions of all individuals. Standard errors (SE) of *b* were approximated by jackknifing. We restricted the range for regression to *d*_*ij*_ = 0–3.0 km, with 3.0 km being the maximum distance of the smallest sampling area (for *F*. *yoponensis* on BCI). For *F*. *insipida*, we calculated *Sp* for all samples collected in the BCNM and for samples exclusively from BCI to compare them to the results of *F*. *yoponensis*. We always refer to the *Sp* statistics when discussing SGS in this manuscript. When comparing SGS among sampling sites or species we classified differences of *Sp* as significant if standard errors (± 2 SE) of *b* did not overlap.

In order to visualize the data for each species, we plotted mean *F*_*ij*_ for n = 5–14 distance classes such that the number of pairwise comparisons within each distance interval was approximately constant. This way, the values for the first distance class do not correspond to the *F*_*1*_ values calculated for the *Sp* statistics. We obtained 95% confidence intervals (CI) by permuting *F*_*ij*_ of all pairs of individuals per distance class 9999 times. Standard errors were assessed by jackknifing for each distance class. All calculations were performed with the program SPAGeDi version 1.3d [37]. In addition, we regressed *F*_*ij*_ over ln(*d*_*ij*_) in R [38] and generated plots with all pairwise comparisons and regression line (S6 Fig).

At larger spatial scales, we used Jost´s *D*_*est*_ and *F*_*ST*_ to determine whether there is genetic differentiation among sampling sites of *F*. *insipida* [39]. Under a model of isolation by distance (IBD) genetic differentiation among populations is expected to increase linearly with distance [40,41]. We used GenAlEx 6.5b3 [42] to calculate pairwise *D*_*est*_ among the sampling sites and SPAGeDI to calculate *F*_*ST*_. In order to test whether *D*_*est*_ increases significantly with distance we conducted a regression of *D*_*est*_ over distance in *R* version 3.0.1. To this end, we calculated a mean value across all pairwise comparisons for the Panamanian sampling sites that were not genetically differentiated (one data point) and used the pairwise comparisons among Panama, Costa Rica, Eastern and Western Peru (six data points). Further, we performed a STRUCTURE analysis [43] to test whether individuals from different Panamanian sampling sites could be assigned to one or more clusters based on multi-locus genotype data. We selected the setting with correlated frequencies as it improves estimates of admixture and left all other parameters at their default values [44]. We used 250,000 MCMC repetitions after a burn-in period of 100,000. To determine the most likely number of clusters *K*, we tested *K* values from 1–10 and carried out 10 runs for each *K*. Afterwards, we followed the procedure suggested by Evanno et al. [45] to select the optimal *K*. With the same settings, we analyzed all samples from Panama, Costa Rica and Peru with 10 repetitive runs for each *K* = 1–7.

Phylogeographic structure can be inferred from the microsatellite DNA data set if allele sizes within a populations or geographic region are more similar than in the overall sample [14]. This was tested by comparing the allele-size based differentiation statistic *R*_*ST*_ with its value after permuting allele sizes within loci (“permuted *R*_*ST*_”—similar to F_ST_) using SPAGeDI with 10,000 permutations. A significant one-sided test establishes the alternative hypothesis of *R*_*ST*_ > “permuted *R*_*ST*_”. Sequential Bonferroni corrections [46] were applied to significance levels of multiple tests.

### Pollen dispersal model

In order to explore the possible role of pollen dispersal limitation in shaping fine scale genetic structure, we designed a computational model using the *R* programming language [38] that allowed us to estimate the number and distance of possible pollen donors for *F*. *insipida* trees in the BCNM. Based on the available census data we included all *F*. *insipida* trees on BCI that were still alive in 2007 and all trees mapped along the shoreline of the islands and peninsulas (n = 342 trees, see S3 Fig).

In the model, each tree undergoes a reproductive cycle that starts at a randomly chosen day of the year. The cycle consists of a receptive phase of six days, a ripening phase of 28 days and a phase of six days during which fig wasps are released and search for fig trees in the receptive phase. The next reproductive cycle starts after a randomly varying period of 228 to 326 days. The model was run for two consecutive cycles for each tree and was repeated ten times. In addition, we repeated the runs with a receptive and release phase of five and seven days, respectively. Trees were classified as potential pollen donors when their release phase overlapped with the receptive phase of another tree, independently of the geographic distance. For each tree we calculated the number of potential pollinators and the Euclidian distance between pollen donor and receptor. Based on these results we estimated the mean number of pollen donors for a number of predefined distance classes. The phenological data for the model was derived from Milton et al. [38,47], Milton [48] and Morrison [24]. We used the R packages “calibrate”, “maptools”, “plotrix” and “sp” [49,50,51,52]. More detailed information on the model is presented as supplementary material (S3 Fig and S2 Methods).

## Results

For each *Ficus* species, we were able to score 7–10 markers with a mean allele number (per locus) of 7.0–10.9 and total allele numbers of 56–109 alleles per species (S2 Table). The number of loci differs between individuals because some of the SSRs did not amplify across all species, had a peak pattern that we could not score reliably, or an excess of null alleles. Null alleles were present in two and three markers for *F*. *citrifolia* and *F*. *obtusifolia*, respectively (S2 Table) and markers with null allele frequencies > 0.2 were omitted from the data analysis.

Each sampled individual had a unique multi-locus genotype. All of the species exhibited high levels of heterozygosity (mean *H*_*O*_ = 0.58–0.79) and low inbreeding coefficients (mean *F*_*IS*_ = -0.056–0.054), except for *F*. *obtusifolia*, which exhibited a relatively high *F*_*IS*_ of 0.146. *Ficus obtusifolia* had an excess of homozygosity in 4 (of 7) loci. Because *F*_*IS*_ is not uniformly high across all loci, we interpret deviations from HWE in the 4 loci as the result of allelic drop-out rather than inbreeding, which would affect all loci. Allele dropout is commonly encountered in microsatellite markers that were not developed for the focal species.

### Spatial genetic structure

For *F*. *insipida*, we found low but significant SGS in the BCNM (*Sp* = 0.003 ± 0.002, p = 0.011, Table 2) and detected comparable values at PLR (*Sp* = 0.0054 ± 0.0052, p < 0.001). Along PLR, SGS was significant in adults (*Sp* = 0.0049 ± 0.0046, p = 0.020), but only marginally significant in saplings (*Sp* = 0.0037 ± 0.0074, p = 0.055).

**Table 2:** Estimates of SGS based on the *Sp* statistics. With *b*: slope of the regression with the correlation coefficient *r* ^*2*^ and *F*_*(1)*_ = kinship coefficient for the first distance class (d < *200*–*300 m*).

Species	Sampling location	*N*	*F_(1)_*	*b* (For *dij* < 3 km)	*r* ^2^	*Sp ± 2SE* *	*P*
*F. insipida*	BCNM	190	0.006	-0.0034	0.001	0.0034 ± 0.0017	0.0113
	- only BCI	88	0.004	-0.0009	0.000	0.0009 ± 0.0026	0.2334
	- only shoreline	102	0.013	-0.0069	0.003	0.0070 ± 0.0049	0.0029
	PLR	155	0.019	-0.0053	0.002	0.0054 ± 0.0052	< 0.001
	- only adults	67	0.020	-0.0048	0.002	0.0049 ± 0.0046	0.020
	- only saplings	73	0.014	-0.0037	0.001	0.0037 ± 0.0074	0.055
*F. citrifolia*	BCNM	62	0.032	-0.0128	0.017	0.0133 ± 0.0083	< 0.001
*F. obtusifolia*	BCNM	59	0.097	-0.0281	0.034	0.0311 ± 0.0228	< 0.001
*F. yoponensis*	BCI	37	0.014	-0.0082	0.005	0.0083 ± 0.0124	0.032

* Standard errors (SE) are based on jackknifing

For the other three fig species we found significant SGS in the BCNM as well. The *Sp* statistics revealed strongest SGS for the strangler fig *F*. *obtusifolia* (*Sp* = 0.031 ± 0.023, p < 0.001) compared to *F*. *citrifolia* (*Sp* = 0.013 ± 0.008, p < 0.001), *F*. *yoponensis* (*Sp* = 0.008 ± 0.012, p = 0.032) and *F*. *insipida*. When comparing *Sp* based on the overlap of 2SE, only *F*. *obtusifolia* and *F*. *insipida* showed significant differences with much stronger SGS in *F*. *obtusifolia*. When we restricted analysis for *F*. *insipida* to trees growing on BCI to better compare it to *F*. *yoponensis*, we found no significant SGS (*Sp* = 0.001 ± 0.003, ns). To exclude the possible of null alleles affecting these results, we repeated the analysis by omitting markers with null alleles (S2 Table). This did not alter the results significantly.

**Fig 2:**
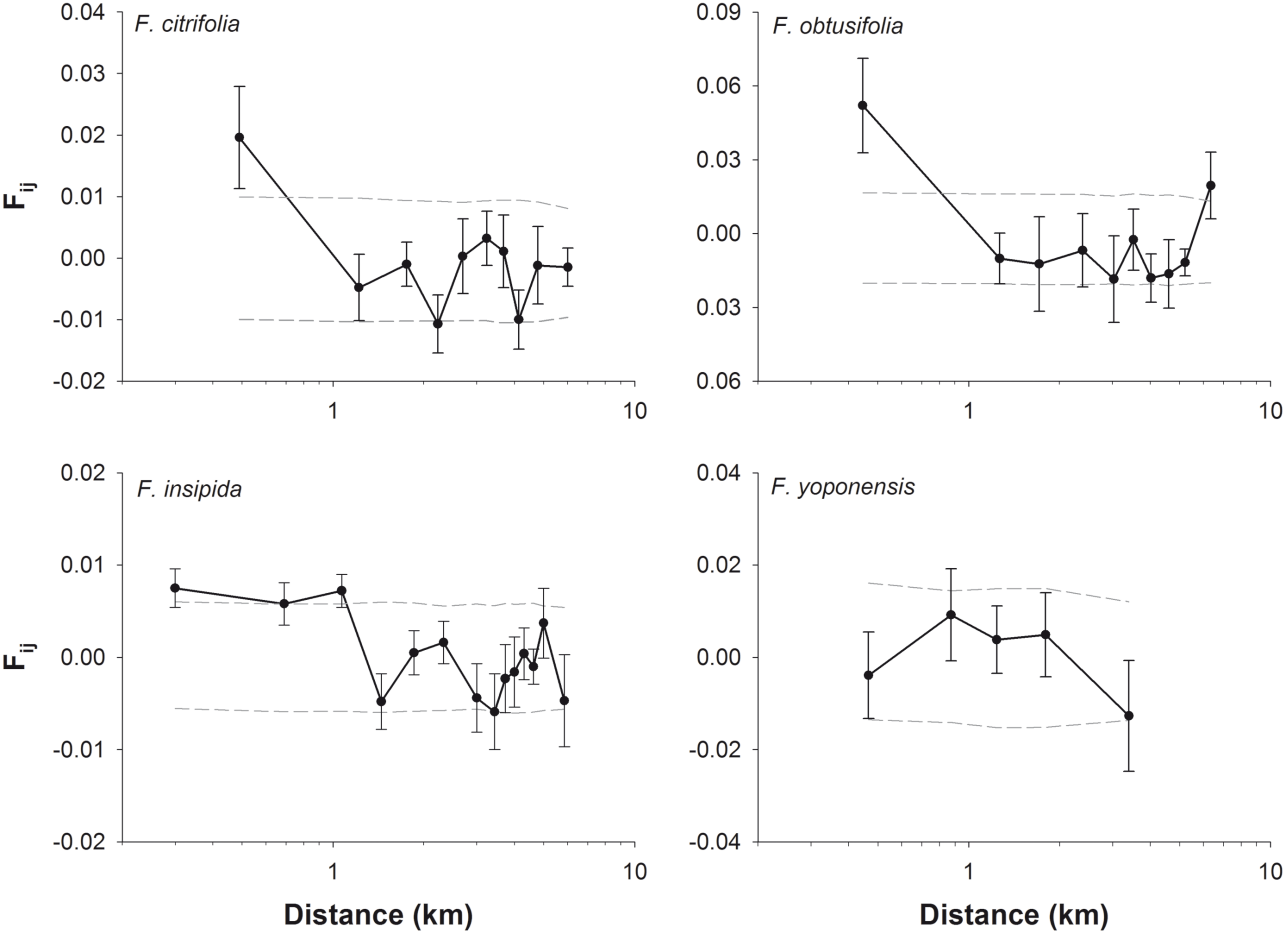
Average kinship over distance for the investigated fig species. Pairwise kinship is determined by Loiselle´s kinship coefficient (*F*_*ij*_) for *F*. *citrifolia* (n_ind_ = 62, n_comp_ = 236), *F*. *obtusifolia* (n_ind_ = 59, n_comp_ = 214), *F*. *insipida* (n_ind_ = 190, n_comp_ = 1795) and *F*. *yoponensis* (n_ind_ = 37, n_comp_ = 133) Dotted lines represent 95% confidence interval. Depending on the pairwise kinship, scales on the y axis vary among graphs. (n_ind_ = number of sampled individuals, n_comp_ = number of pairwise comparisons per distance class).

The autocorrelation analysis largely supported the finding of significant SGS and indicated that pairwise kinship was higher than expected under conditions of random pollen and seed dispersal (reflected by the 95% CI) for distance classes up to 1 km in *F*. *insipida* (Fig 2). For both strangler figs the pairwise kinship coefficient of the first distance class (mean pairwise distance < 500 m) was significant. In *F*. *yoponensis*, the autocorrelations was not significant, but *F*_*ij*_ decreases with increasing distance as well except for the first distance class.

### Pollen dispersal model

**Fig 3:**
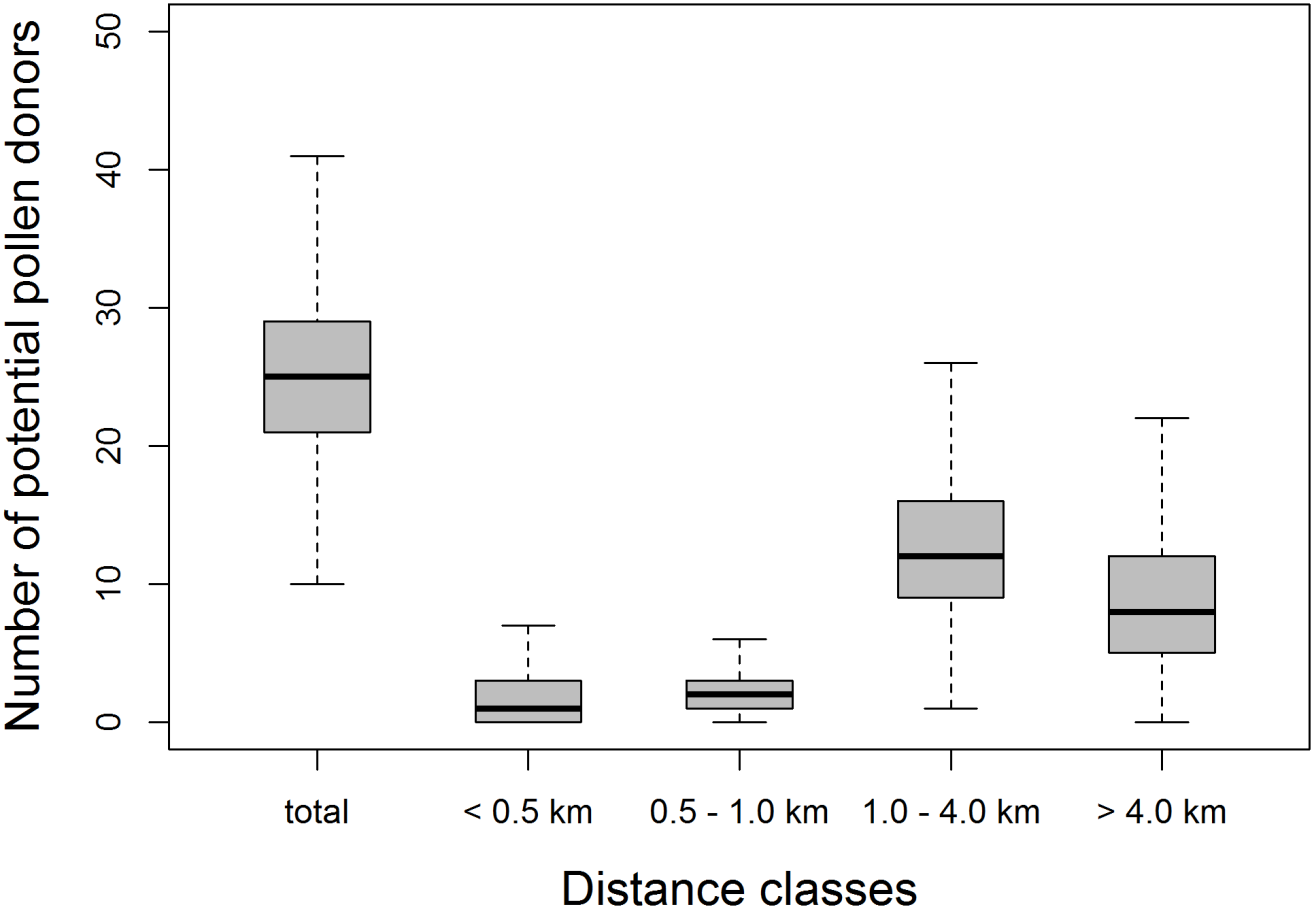
Simulation results of the pollen dispersal model. Results are based on simulations with a receptive and release phase of six days. Boxplots represent the number of trees that can act as potential pollen for each receptive *F*. *inspida* tree at the given distance classes. Results are based on 10 runs.

When the receptive and release phase in our model were set to six days, we found that 48 out of 342 *F*. *insipida* trees had no potential mate within a radius of 500 m (mean number of mating partners < 500 m: 1.7 ± 1.0). On average, 4.0 ± 2.1 potential pollen donors could be encountered within a radius of 1 km in our simulations (Fig 3). In total, each receptive tree could potentially receive pollen from 25.8 ± 2.3 trees in our simulation. Mean distance between potential mating partners was 3.0 ± 0.5 km. If the receptive and release phase lasted for five or seven days, mean number of pollen donors decreased by 13% and increased by 14%, respectively (S4 Fig).

### Genetic differentiation of *F*. *insipida* at regional and biogeographic spatial scales

**Fig 4:**
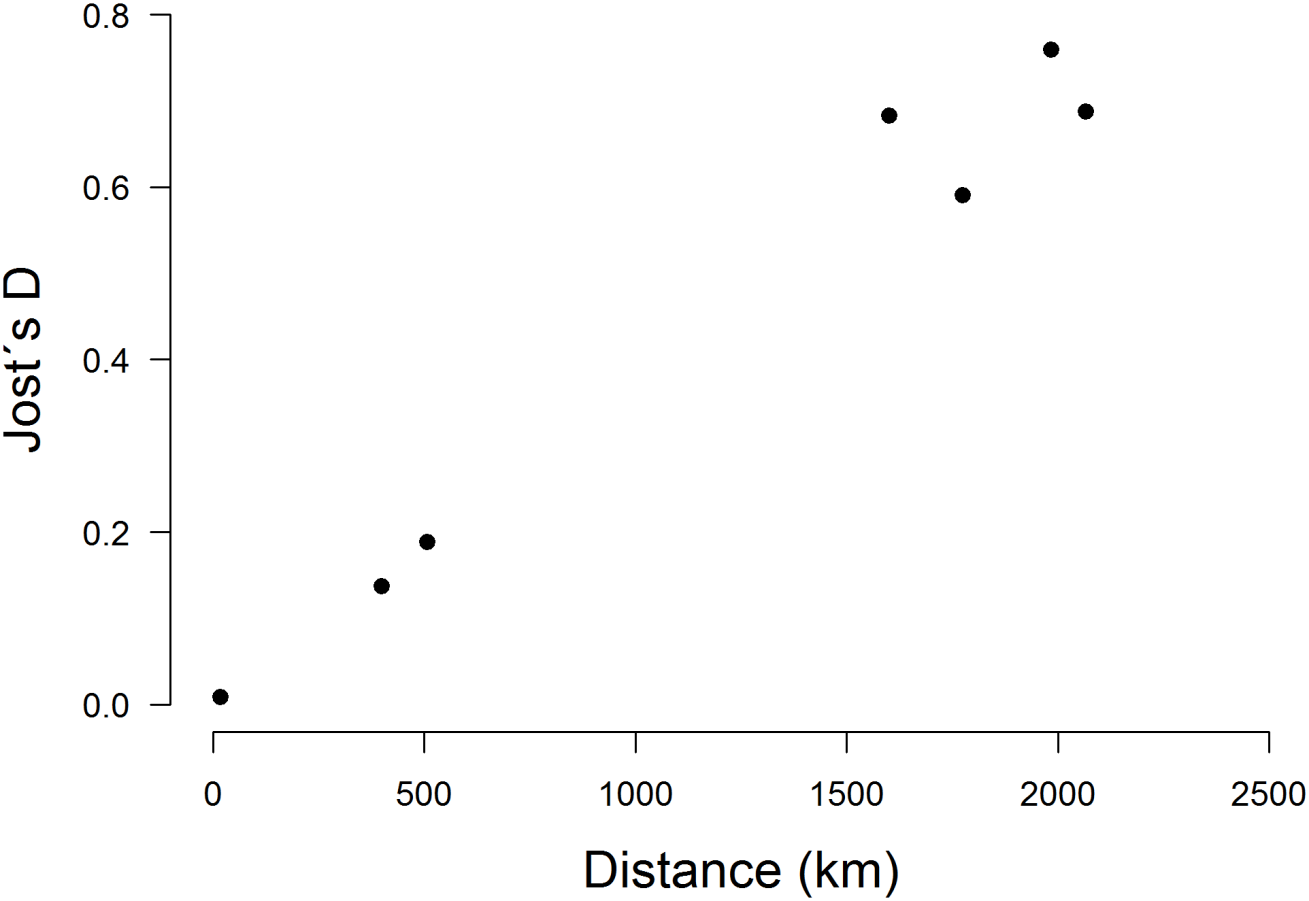
Genetic differentiation among *F*. *insipida* sampling sites located in Panama, Costa Rica and Peru. Genetic differentiation given as Jost´s *D*_*est*_ increases linearly with spatial distance as expected under IBD or IBB (adjusted R^2^ = 0.962, p < 0.001). As there was no population substructure among Panamanian sampling sites, they are represented by one data point at short distance while the other data points are pairwise comparisons among Panama, Costa Rica, Western and Eastern Peru.

At the regional scale, genetic differentiation among sampling sites located along the Panama Canal was low (*D*_*est*_ = 0.009 ± 0.006, *F*_*ST*_ = 0.006 ± 0.004) and did not increase with distance. The STRUCTURE analysis detected only one cluster in this entire area. In contrast, at the biogeographic scale (Costa Rica to Peru) we found a strong linear correlation of *D*_*est*_ over linear Euclidian distance (adjusted R^2^ = 0.962, p < 0.001, Fig 4, corresponding *F*_*ST*_ in Table 3). The most likely number of clusters of the corresponding STRUCTURE analysis was *K* = 2 separating the samples from Peru and Central America. However, the Evanno method has a strong bias to identify *K* = 2 as the most likely number due to the low variance of *K* = 1 [53]. Thus, we also assessed the second most likely number of clusters, which was *K* = 5. In that case, the samples from Peru and Costa Rica clustered separately while individuals from Panama were jointly assigned to three clusters (S2 Fig).

**Table 3:** Pairwise measures of genetic differentiation between *F*. *insipida* populations. *F*_*ST*_ (above diagonal) and *R*_*ST*_ (below diagonal). If *R*_*ST*_ > permuted *R*_*ST*_, it is indicative for the contribution of mutations to population differentiation (for p < 0.05 after correction for multiple testing indicated by bold numbers).

	Panama	Costa Rica	Western Peru	Eastern Peru
Panama	-	0.121	0.261	0.281
Costa Rica	0.129	-	0.324	0.323
Western Peru	**0.441**	**0.500**	-	0.049
Eastern Peru	**0.533**	**0.552**	0.030	-

The phylogeographic analysis based on microsatellite size variation showed significant difference between *R*_*ST*_ and permuted *R*_*ST*_ only in comparisons between Central America (Costa Rica or Panama) and Peru (P < 0.01) (Table 3). There was no significant contribution of allele size mutation to population differentiation within Central America or within Peru.

## Discussion

We collected nuclear microsatellite data to estimate genetic structure at different spatial scales in the common free-standing fig *F*. *insipida*. Significant genetic differentiation among Mesoamerican and Amazonian sites indicates that pollen dispersal is restricted over biogeographic scales. At the local scale we detected weak but significant SGS in *F*. *insipida* as well as in three other sympatric fig species from both New World subgenera *Pharmacosycea* (free-standing figs) and *Urostigma* section *Americana* (strangler figs). Our model results were consistent with previous genetic studies [7] that showed that pollen dispersal routinely exceeds the area within which we estimated SGS. Thus, our results suggest that local scale SGS results from relatively limited seed dispersal in Neotropical figs. In the following, we explore key processes that influence the formation of SGS in Neotropical figs and relate SGS at the local scale to patterns of geographic differentiation.

Figs exhibit the highest pollination distances known in insect-pollinated plants [3]. In Panama, fig wasps often need to travel hundreds to thousands of meters to encounter a receptive conspecific host plant. Individual figs routinely receive pollen from multiple genetically distinct pollen sources that can be dozens of kilometers apart [7]. Asynchronous mating further reduces the frequency of nearest neighbor pollination. In accordance with this, we observed high *H*_*O*_ and low *F*_*IS*_ values in our study which indicate high levels of random mating. Our model results using data from *F*. *insipida* are consistent with Nason et al.´s [7] findings with stranglers: only very few pollen donors are routinely located among the nearest neighbors of a receptive fig tree: For 44% of the modeled trees 0–1 potential pollen donors were located within a radius of 500 m, while a much larger number of pollen donors was located beyond 1 km (84.5% of all potential fathers in our model), far exceeding the spatial scale of the fine scale SGS in our study. Our model likely underestimates the number of pollen donors at larger distances because it is restricted to the area of BCNM. As we do not integrate wind speed and direction, we probably overestimate the number of pollen donors at short distances. Nevertheless, our estimate demonstrates that close neighbor mating plays a minor role in pollen mediated gene flow and thus, is unlikely to generate local scale SGS. Our pollination model is likely to apply broadly to Neotropical fig species, most of which occur at even lower densities than *F*. *insipida* yet have similar asynchronous flowering patterns.

While long distance pollen dispersal is a common feature in Neotropical figs, SGS patterns may vary predictably among Neotropical *Ficus* species for other reasons, including life history differences associated with fruit size, and differences in habitat requirements for seedling establishment, both of which we explore in the following paragraphs. As any measure of SGS will only provide a temporally and spatially restricted snapshot, we also discuss the potential influences of colonization history and fluctuations in population density on SGS of the studied fig populations.

### Regeneration ecology, colonization history and SGS

While fig species mostly occur at low densities, free-standing figs might occur—temporally and/or locally restricted—at higher densities, especially in former disturbed areas and light gaps [26]. The intensity of SGS can depend on the degree to which large light gaps are colonized by seeds from multiple sources [54]. The relatively low SGS observed in *F*. *insipida* in our study, coupled to the fact that SGS is not significant even in saplings, suggests that light gaps are being seeded by multiple maternal sources. As free-standing figs are most dominant in early successional stages of forests, colonization history has to be considered as well. The northwestern part of BCI experienced a long history of agricultural use and the forest only began to regenerate about 150 years ago. Almost all *F*. *insipida* and *F*. *yoponensis* trees on BCI are restricted to this secondary forest [26]. Most free-standing *Ficus* trees established in early successional stages and appear to be the same age on BCI. As the forest matured, further recruitment was impeded by low light conditions under the canopy cover and, currently, sapling *Ficus* trees are extremely rare in BCI´s closed canopy forest [26,55]. Regular census data since 1973 has shown a drastic decline of both studied free-standing fig species on the island in recent years (Albrecht et al. unpublished data, [24]) indicating that the trees have reached the end of their natural life spans. Thus the absence of SGS in *F*. *insipida* on BCI (S5 Fig) could be attributed to the fact that trees established almost simultaneously from multiple seed sources that were likely growing at the edge of the agricultural fields. Initial patterns of SGS would then be weakened by demographic thinning [56]. In contrast, seeds of strangler figs mostly establish in the canopies of other host trees, and they should encounter evenly scattered germination sites within intact forest. A relatively random distribution of fig trees at low density, such as is observed in the stranglers, should increase SGS because seed shadow overlap is expected to be relatively low [14]. We expect that future studies will find similar differences in the intensity of SGS between strangler and free-standing figs due to their fundamentally different requirements for seedling establishment.

### Influence of frugivore size and dispersal on SGS

While howler monkeys and frugivorous birds visit fruiting figs trees during daytime [57,58], in BCNM, fruit-eating bats of the family Phyllostomidae disperse more than 80% of the seeds of the studied fig species [23,59]. Bat-mediated seed dispersal distances are unknown due to the observational challenges posed by the high mobility and nocturnal lifestyle of bats. Although fruit-eating phyllostomid bats are generally regarded as potential long distance seed dispersers [60,61], several factors may limit their effective dispersal distances. After picking a fruit, these bats usually fly to temporary feeding roosts located within 50–200 m of the fruiting tree [62]. This behavior appears to be a response to predators that also congregate at ripe fig trees [62]. Ingested seeds are not likely to travel far as bats have short gut passage time for seeds (< 30 min) [63] which leaves germination rate of seeds largely unaffected [60]. Based on this, we expect that most fig seeds will be dropped below or near these temporary feeding roosts close to the maternal fig tree. Larger fig fruits are generally consumed by bat species with larger body size [64,65,66] that are more likely to disperse seeds over longer distances due to their higher mobility. According to that rationale, *F*. *insipida* and *F*. *obtusifolia* should be dispersed over larger distances and exhibit lower SGS (see Table 1 for fruit sizes). However, we found the lowest SGS in *F*. *insipida* and the highest in *F*. *obtusifolia* (Table 2). We conclude that measurable differences in seed dispersal distances are either not significant among fig species or that their effects on local SGS are overwhelmed by other factors. While encountering seeds dispersed by fruit-eating bats is not an easy task, genetic parentage analyses of fig seeds deposited under feeding roosts could provide the first empirical data on seed dispersal distance by fruit-eating bats.

Our results of low but significant SGS are also consistent with those of Nazareno et al. [12] who studied SGS in populations of *F*. *citrifolia* and *F*. *eximia*. It is also interesting to note that despite the extraordinary pollen dispersal distances documented in figs, other tropical species that are pollinated by insects and dispersed by animals show similar levels of SGS based on the *Sp* statistics as the figs from our study (compare [2,67]). We suggest that the observed SGS values in our study are primarily shaped by differences in tree density, and the interplay between regeneration strategies, colonization history, and average seed dispersal distances. However, while SGS measured with the *Sp* statistics is generally considered robust across different sampling schemes [14], we recognize that SGS can also be influenced by population dynamics and idiosyncratic population histories [68] that may obscure more deterministic differences in SGS caused by life history.

### Regional and phylogeographic genetic structure of *F*. *insipida*

At the regional scale within the Panama Canal watershed we detected no population substructure for *F*. *insipida* despite the spatial limitations of seed dispersal that likely generated SGS at the local scale. This is consistent with prior estimates of breeding area based on pollen dispersal studies for strangler figs [7]. At the biogeographic scale, we found that populations were genetically distinct and genetic differentiation increased with distance as predicted under conditions of isolation by distance (IBD) and/or isolation by geographic barriers (IBB) [69] (Table 3). The geographic barriers separating our population samples from Costa Rica to Peru include the northern Andes, the dry Llanos region of northern South America, and various xeric and montane barriers between the Panamanian and Costa Rican sites. While the summary statistics *F*_*ST*_, *R*_*ST*_ and *D*_*est*_ only permit a *post hoc* interpretation, the phylogeographic structure of *F*. *insipida* detected between our Central and South American samples is consistent with the previously published cpDNA phylogeography [18] of the same species. Because the phylogeographic structure was stronger in the maternally transmitted cpDNA than in ITS, Honorio Coronado and colleagues [18] suggested that the geographic barriers associated with the northern Andes posed a greater barrier for seed than pollen dispersal. This pattern may have other explanations. The haploid, uniparentally transmitted chloroplast genome has a lower *N*_e_ (1/4 the N_e_ of nuclear markers) and genetic drift therefore plays a much stronger role in cpDNA markers. Chloroplast capture from congeneric species is also possible, and has been demonstrated in *Ficus* and other tree taxa [70,71]. The absence of ITS variation between Panama and Amazonian *Ficus* [18] is among the lowest levels of divergence found in lowland trees sampled on either side of the Andes [72,73,74,75] and is most similar to species that have relatively recently dispersed around the Andes [76]. This suggests fig seed dispersal and/or colonization ability (especially in drier habitats) has allowed *F*. *insipida*, and perhaps other fig species, to overcome the geographic barriers that have caused vicariance in many other tree species.

## Conclusions

Neotropical fig species exhibit local spatial genetic structure despite frequent long pollen dispersal distances. The most likely underlying cause is limited seed dispersal, with fruit-eating Phyllostomid bat species being the most likely seed dispersers of the studied *Ficus* species in BCNM. In addition, different regeneration strategies of free-standing vs. strangler figs may cause differences in the degree of SGS. Nevertheless, gene flow over large distances is maintained via pollen dispersal and our study of meso-scale genetic structure in *F*. *insipida* confirmed that breeding populations cover large areas such as the entire watershed of the Panama Canal. Yet, at biogeographic spatial scales, genetic differentiation among regions becomes apparent, and is likely caused by a combination of IBD and IBB. Further studies are needed to test the emerging pattern of greater gene flow in monoecious than in dioecious species (this study and [11,12,77]). Importantly, species-specific traits (e.g. requirements for seedling recruitment) as well as the behavior of both pollen and seed dispersal agents have to be considered when predicting the spatial extent of gene flow in figs and other tree taxa.

## Supporting Information

S1 Dataset SSR raw data for all individuals. (XLSX)

S1 Fig *Ficus* sampling in the Barro Colorado Nature Monument, Panama (top rows) and along Pipeline Road (bottom row) for the assessment of SGS. Shown are all sampled individuals of *F*. *insipida*, *F*. *yoponensis*, *F*. *citrifolia* and *F*. *obtusifolia*.
(PNG)

S2 Fig Assignment of samples from Panama (n = 429), Costa Rica (n = 27) and Peru (n = 27) to cluster with the Structure software. The most likely number of cluster was *K* = 5 where samples from Peru and Costa Rica were assigned to separate clusters while samples from Panama were jointly assigned to three clusters. The graph was based on the run that had the highest Ln P(D) value with *K = 5*.
(PNG)

S3 Fig Map of the BCNM with all *F*. *insipida* trees mapped on BCI and the surrounding islands and peninsulas that were integrated in the pollen flow model (n = 342, all dots). Here, tree 54 (blue dot) is in its receptive phase and all trees highlighted in red release fig wasps at a given day. Blue circles around tree 54 indicate the distance classes (< 500 m, 501–1.000 m, 1.001–4000 m).
(PNG)

S4 Fig Number of trees that can act as potential pollen donors in the pollen dispersal model for *F*. *inspida*. Boxplots are based on 10 runs where the release and receptive phase last for five (A) and seven (B) days each.
(TIF)

S5 Fig Average kinship over distance for the *F*. *insipida* along the shoreline of BCNM (n_ind_ = 102, n_comp_ = 515) and on BCI (n_ind_ = 88, n_comp_ = 478). Pairwise kinship is determined by Loiselle´s kinship coefficient (*F*_*ij*_). Dotted lines represent 95% confidence interval. Depending on the spatial scale the x axes vary between graphs. (n_ind_ = number of sampled individuals, n_comp_ = number of pairwise comparisons per distance class).
(TIF)

S6 Fig Regression of *Fij* over ln(*d*_*ij*_) for the four sampled species. Regression in based on all pairwise comparisons within a radius of 3 km. Regression was performed in R using a linear model. Within each graph, r2, the regression slope b and p are indicated (number of pairwise comparisons for each species: Fcit = 962, Fins = 7764, Fobt = 761 and Fyop = 664).
(TIFF)

S1 Methods Methods for chloroplast SSRs. With Table A: Allele length for the tested cpSSRs from Weising & Gardner (1999).
(DOCX)

S2 Methods Specifications of the pollen dispersal model. (DOCX)

S1 Table Sampling sites of *F*. *inspida* in Panama, Costa Rica and Peru with the number of samples per plot (n) and the spatial coordinates of the *locality*. (DOCX)

S2 Table Characterization of the microsatellite markers for *Ficus* samples collected in the Barro Colorado Nature Monument. For each marker, we provide the percentage of missing data, the number of alleles (N_a_), the expected heterozygosity (H_e_) and the inbreeding coefficient (F_IS_).
(DOCX)
