# Supplementary figures and images for: Spatial Scales of Genetic Structure in Free-Standing and Strangler Figs (Ficus, Moraceae) Inhabiting Neotropical Forests
Source: PLoS One. 2015 Jul 30;10(7):e0133581. doi: 10.1371/journal.pone.0133581 (PMC4520606; doi:10.1371/journal.pone.0133581)

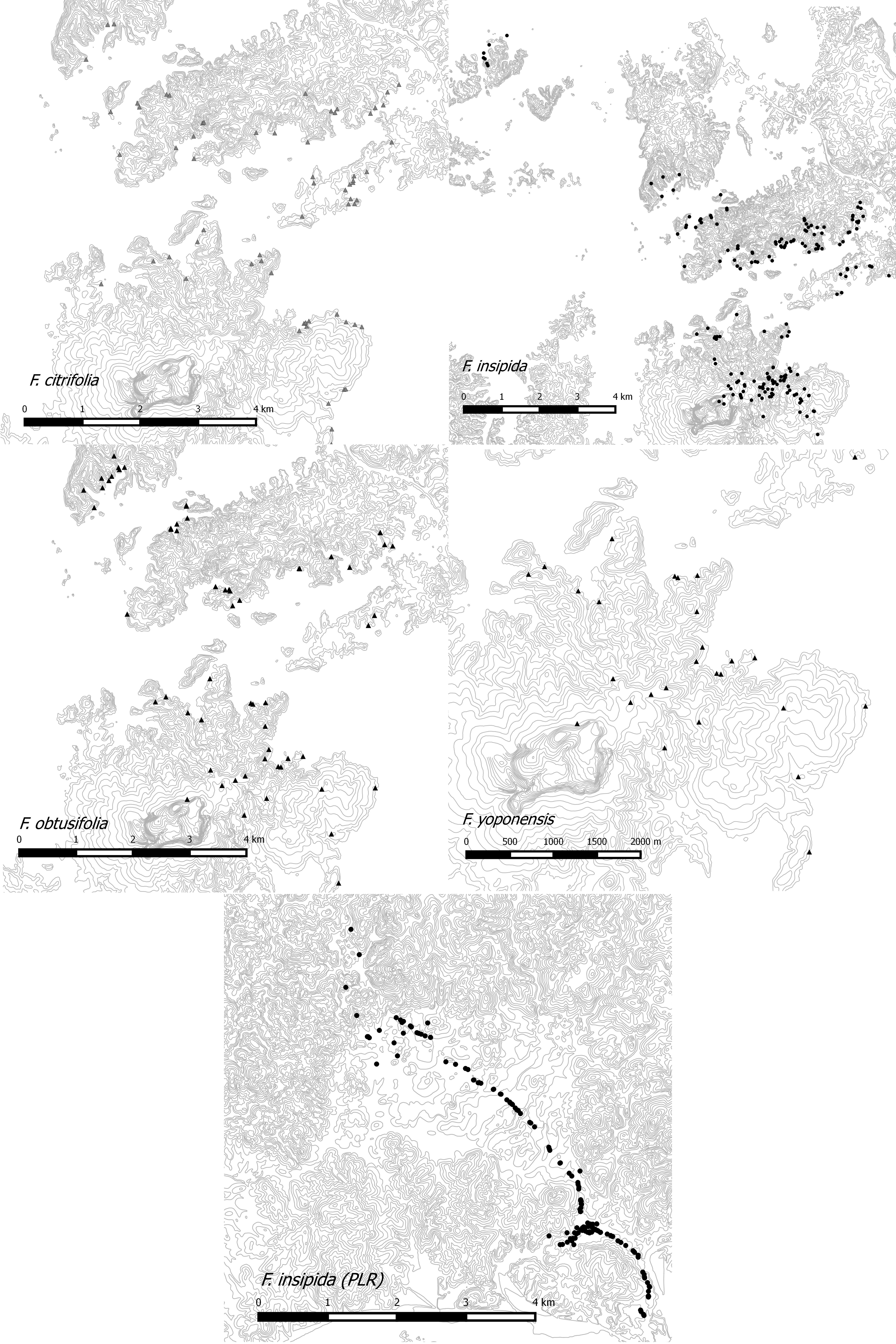

Supplement: S1 Fig — Shown are all sampled individuals of F. insipida, F. yoponensis, F. citrifolia and F. obtusifolia. (PNG) [file pone.0133581.s002.png]

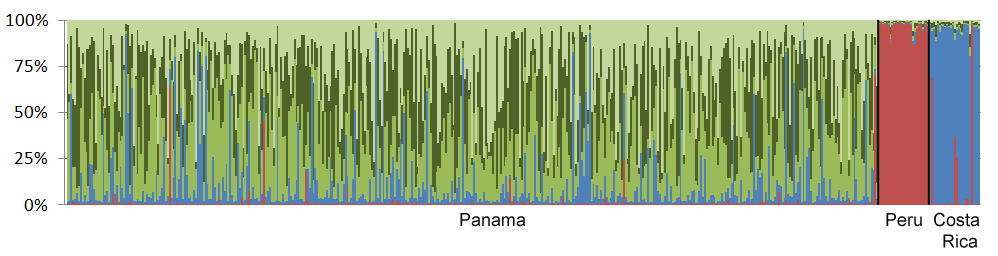

Supplement: S2 Fig — The most likely number of cluster was K = 5 where samples from Peru and Costa Rica were assigned to separate clusters while samples from Panama were jointly assigned to three clusters. The graph was based on the run that had the highest Ln P(D) value with K = 5. (PNG) [file pone.0133581.s003.png]

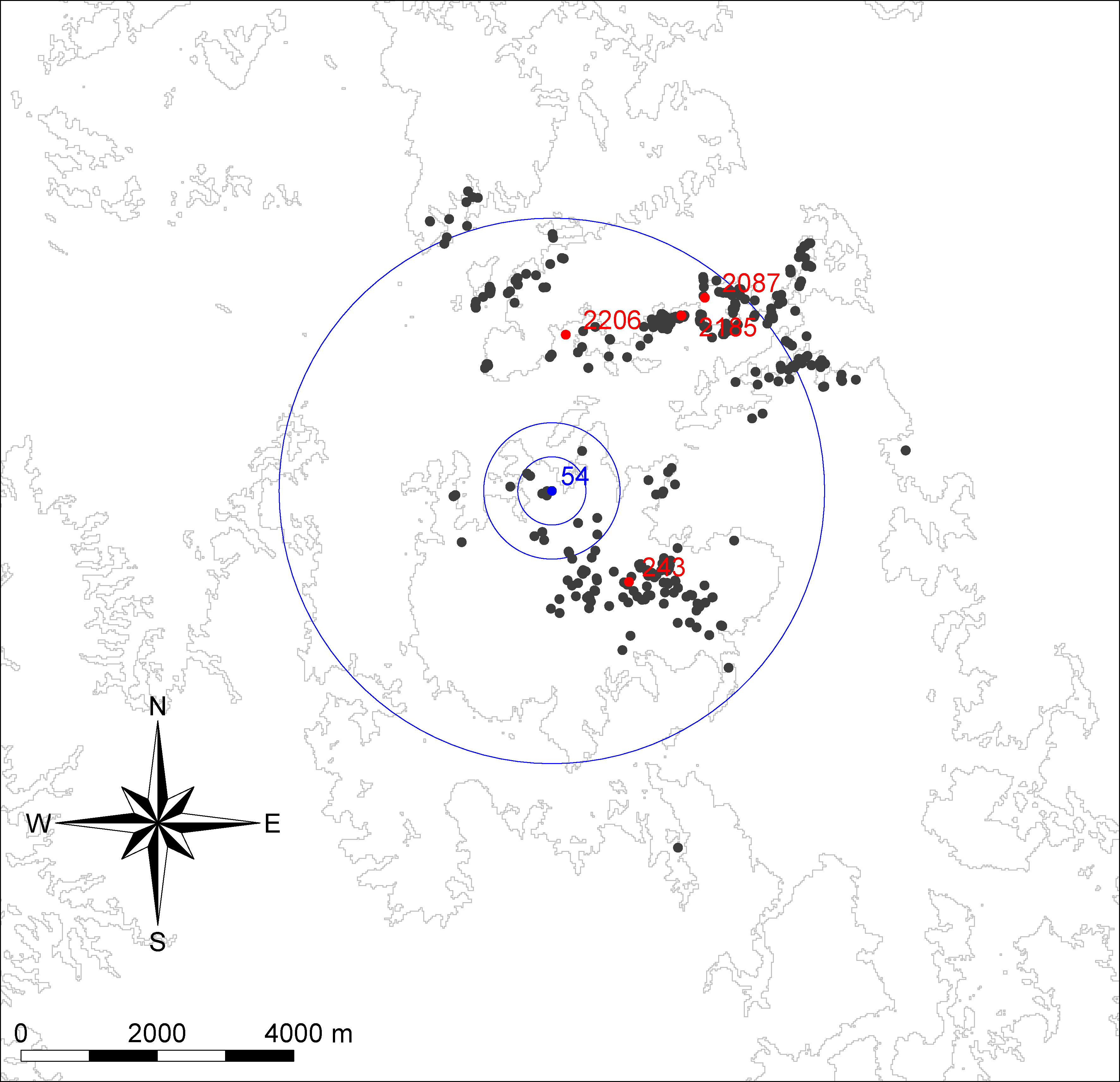

Supplement: S3 Fig — Here, tree 54 (blue dot) is in its receptive phase and all trees highlighted in red release fig wasps at a given day. Blue circles around tree 54 indicate the distance classes (< 500 m, 501–1.000 m, 1.001–4000 m). (PNG) [file pone.0133581.s004.png]

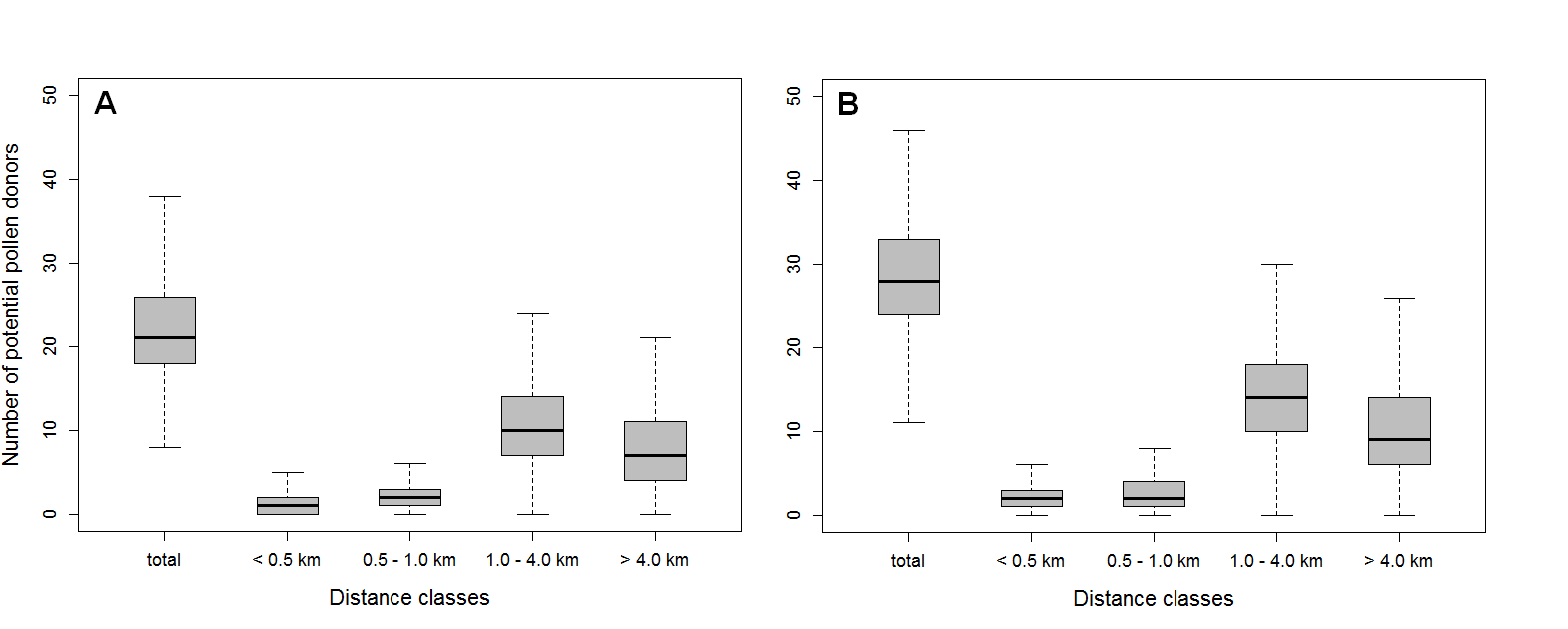

Supplement: S4 Fig — Boxplots are based on 10 runs where the release and receptive phase last for five (A) and seven (B) days each. (TIF) [file pone.0133581.s005.tif]

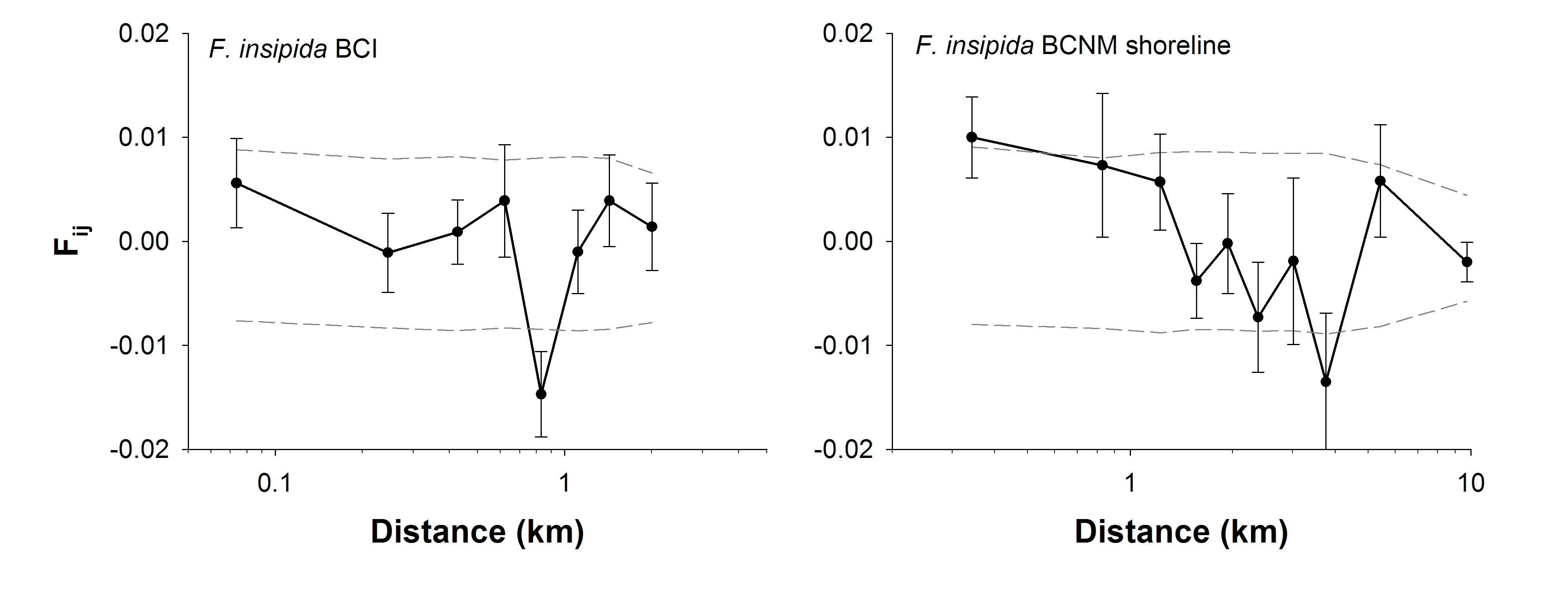

Supplement: S5 Fig — Pairwise kinship is determined by Loiselle´s kinship coefficient (F ij). Dotted lines represent 95% confidence interval. Depending on the spatial scale the x axes vary between graphs. (nind = number of sampled individuals, ncomp = number of pairwise comparisons per distance class). (TIF) [file pone.0133581.s006.tif]

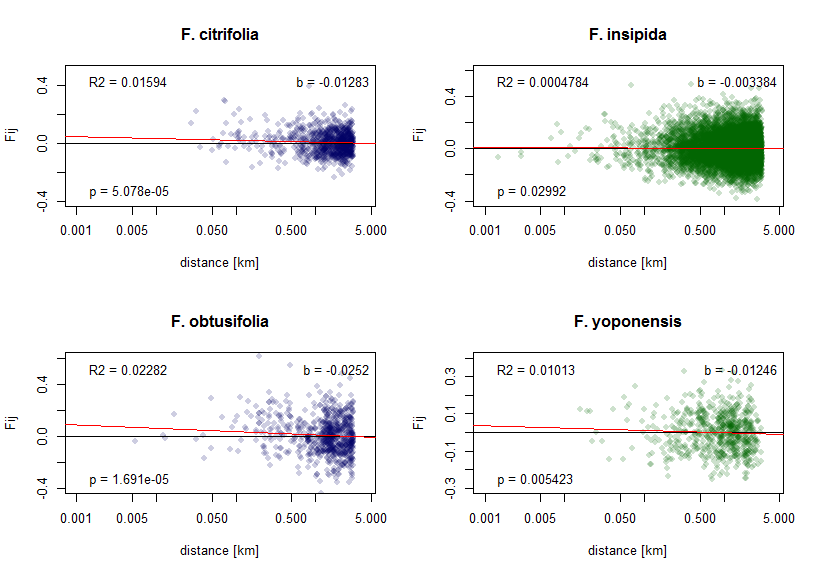

Supplement: S6 Fig — Regression in based on all pairwise comparisons within a radius of 3 km. Regression was performed in R using a linear model. Within each graph, r2, the regression slope b and p are indicated (number of pairwise comparisons for each species: Fcit = 962, Fins = 7764, Fobt = 761 and Fyop = 664). (TIFF) [file pone.0133581.s007.tiff]
