# Supplementary material for: Spatial Scales of Genetic Structure in Free-Standing and Strangler Figs (Ficus, Moraceae) Inhabiting Neotropical Forests
Source: PLoS One. 2015 Jul 30;10(7):e0133581. doi: 10.1371/journal.pone.0133581 (PMC4520606; doi:10.1371/journal.pone.0133581)
Supplement: S1 Methods — With Table A: Allele length for the tested cpSSRs from Weising & Gardner (1999). (DOCX) [file pone.0133581.s008.docx]

**S1 Methods: Methods for chloroplast SSRs**

We tested a number of maternally inherited chloroplast markers in order to obtain additional information on seed dispersal distances. We tested for polymorphism in chloroplast microsatellite loci (cpSSR), including seven published by Weising and Gardner (1999) using 24 samples of *F. insipida* and 16 samples of *F. obtusifolia* and *F. citrifolia* originating from Costa Rica, Panama and for *F. insipida* also from Peru*.* While most of the markers amplified in F. insipida, amplification was not suffiecient to evaluate the data for the two strangler fig species in most cases. None of the investigated markers was polymorphic (except for one allele in on individuals for ccmp2) in the tested species, consistent with previously observations of paucity of variation of cytoplasmic genetic markers in figs (see Herre *et al.*, 1996).

**Table A: Allele length for the tested cpSSRs from Weising & Gardner (1999).** All but ccmp2 were monomorphic in *F. insipia*. The few markers that were amplified successfully in *F. citrifolia* and *F. obtusifolia* were monomorphic within the species and only exhibited a different allele in ccmp2 across species (na: no amplification).

|  | *F. insipida* | *F. citrifolia* | *F. obtusifolia* |
| --- | --- | --- | --- |
| ccmp1 | 143 | na | na |
| ccmp2 | 270/275 | 264 | na |
| ccmp3 | 118 | na | na |
| ccmp4 | 121 | na | na |
| ccmp6 | 115 | 115 | 115 |
| ccmp7 | 128 | na | na |
| ccmp10 | 121 | 121 | na |

**References**

Herre EA et al. (1996) Molecular phylogenies of figs and their pollinator wasps. J. Biogeogr. 23:521-530

Weising K, Gardner RC (1999) A set of conserved PCR primers for the analysis of simple sequence repeat polymorphisms in chloroplast genomes of dicotyledonous angiosperms. Genome 42:9-19
