# Supplementary material for: Spatial Scales of Genetic Structure in Free-Standing and Strangler Figs (Ficus, Moraceae) Inhabiting Neotropical Forests
Source: PLoS One. 2015 Jul 30;10(7):e0133581. doi: 10.1371/journal.pone.0133581 (PMC4520606; doi:10.1371/journal.pone.0133581)
Supplement: S2 Methods — (DOCX) [file pone.0133581.s009.docx]

**S2 Methods: Specifications of the pollen dispersal model**

STATE VARIABLES AND SCALES

We included all *F. insipida* trees in the model that were censused on BCI and the surrounding islands and peninsulas (Fig S3). For BCI, the data is based on a census that was first conducted as a complete census from 1985 - 1989. We included all trees that were reported to be alive during the last complete census in 2007 (Albrecht, Kalko & Handley, unpubl. data).

The development cycle of figs and fig wasps is based on data from Milton et al. (1982) and Milton (1991). Each tree undergoes four consecutive phases of specific lengths: RECEIVING, DEVELOP-MENT, RELEASE and REBUILD. In the RECEIVING phase (6 days) trees are receptive for pollen of other trees. During the subsequent DEVELOPMENT phase, action is suspended for 28 days. The RELEASE of 6 days length mimics the phase of *Ficus* in which fig wasps hatch from fruits and search for blooming flowers of other trees of their host species. Instead of modeling wasps as agents, a hit rate of 100% percent between RELEASING and RECEIVING trees is assumed. Like the DEVELOP-MENT phase, REBUILD is also technically a pause. While the other phases have fixed lengths, its length is designed to individually vary between 228 and 326 days. To calculate the individual length of the REBUILD phase, a normally distributed number between 0 – 98 was randomly drawn and added to 228. After the end of the rebuild phase a new cycle for a tree starts, one simulation run consists of two complete development cycles of each tree. To ascertain that trees start in equally distributed development phases, the first day of the RECEIVING phase of each tree is determined by a random sample and could be any day in the first year of the simulation.

PROCESS OVERVIEW AND SCHEDULING

Potential pollinators of a tree are identified by comparing the temporal overlap of its RECEIVING phase to the RELEASE phases of all other trees in the model. Trees with overlapping RELEASE phases get listed as potential pollinators once per overlapping day. Furthermore, the Euclidean distance between the RECEIVING and the RELEASING tree is calculated. The result of each simulation run is a list that contains all potential pollinators with Euclidean distances for all RECEIVING days of each individual tree.

DESIGN and DETAILS

For every simulation run, agents are positioned at the WGS 84 coordinates based on the real census data. While most developmental phases of the trees are fixed, at the initialization of a simulation the first day of the RECEIVING phase is determined to start randomly at a day from 1 to 365 for every agent. The subsequent REBUILD phase is then randomly drawn from 228 and 326 (see description above). The first of the two random factors is included to provide a *Ficus*-like perennial cycle, the second to create individual variability and thus permit interactions between changing agents in the second cycle. The random variables are newly calculated at the start of a simulation run.

ANALYSIS

10 separate simulation runs with new random variables were conducted to suspend artifact interaction between individuals.

For further analysis of the simulation results, four distance classes for the fast attribution of pairwise distances were set as 0 to 500, 501 to 1000, 1001 to 4000 and over 4001 meters distance. For each cycle of an agent, the number of individual pollinators overall, the number of pollinators in each distance class and the average Euclidean distance from the receiver to the releasing agents were calculated.

**References**

Milton K. (1991). Leaf change and fruit production in six neotropical Moraceae species. J. Ecol., 79, 1-26.

Milton K., Windsor D.M., Morrison D.W. & Estribi M.A. (1982). Fruiting phenologies of two neotropical Ficus species. Ecology (Wash. D. C.), 63, 75
