# Supplementary material for: Spatial Scales of Genetic Structure in Free-Standing and Strangler Figs (Ficus, Moraceae) Inhabiting Neotropical Forests
Source: PLoS One. 2015 Jul 30;10(7):e0133581. doi: 10.1371/journal.pone.0133581 (PMC4520606; doi:10.1371/journal.pone.0133581)
Supplement: S1 Table — (DOCX) [file pone.0133581.s010.docx]

**S1 Table**. **Characterization of the microsatellite markers for *Ficus* samples collected in the Barro Colorado Nature Monument**. For each marker, we provide the percentage of missing data, the number of alleles (N_a_), the expected heterozygosity (H_e_) and the inbreeding coefficient (F_IS_). Significant deviations from HWE are indicated by asterisk (* < 0.05, ** < 0.01, *** < 0.001). Null allele frequencies are given as means from the four estimators in Microchecker [30].

|  | ***F. citrifolia* (n = 63)** | | | | | ***F. obtusifolia* (n = 60)** | | | | | ***F. insipida* (n = 190)** | | | | ***F. yoponensis* (n = 37)** | | | |
| --- | --- | --- | --- | --- | --- | --- | --- | --- | --- | --- | --- | --- | --- | --- | --- | --- | --- | --- |
| **Marker** | **Missing**  **data** | ***N_a_*** | ***H_e_*** | ***F_IS_*** | **Null allele**  **frequencies** | **Missing**  **data** | ***N_a_*** | ***H_e_*** | ***F_IS_*** | **Null allele**  **frequencies** | **Missingdata** | ***N_a_*** | ***H_e_*** | ***F_IS_*** | **Missing data** | ***N_a_*** | ***H_e_*** | ***F_IS_*** |
|  |  |  |  |  |  |  |  |  |  |  |  |  |  |  |  |  |  |  |
| FC14 ^a^ | 1.6% | 7 | 0.68 | 0..005 | - | 11.9% | 4 | 0.71 | 0.28** | 0.175 ± 0.092 | 5.3% | 11 | 0.8 | -0.01 | 0.0% | 3 | 0.29 | -0.181 |
| FC22 ^a^ | 1.6% | 12 | 0.89 | 0.119 | - | 1.7% | 12 | 0.85 | 0.15*** | 0.081 ± 0.17 | 3.7% | 16 | 0.9 | 0.042 | 0.0% | 10 | 0.83 | 0.151 |
| FP21 ^a^ | 3.2% | 7 | 0.81 | -0.03* | - | - | - | - | - | - | 3,7% | 5 | 0.5 | 0.064 | 0.0% | 8 | 0.57 | -0.132 |
| FP22 ^a^ | 8.1% | 6 | 0.75 | 0.066 | 0.063 ± 0.017 | 6.8% | 6 | 0.60 | 0.06 | - | 3.2% | 8 | 0.6 | 0.038 | 10.8% | 7 | 0.79 | 0.153 |
| FP25 ^a^ | 1.6% | 7 | 0.66 | 0.019 | - | - | - | - | - | - | - | - | - | - | - | - | - | - |
| FP57 ^a^ | 0.0% | 22 | 0.93 | 0.068 | - | 15.3% | 18 | 0.92 | 0.16*** | 0.129 ± 0.111 | - | - | - | - | - | - | - | - |
| FinsA1^b^ | 1.6% | 7 | 0.72 | -0.06 | - | 1.7% | 4 | 0.50 | 0.14 | - | 0.5% | 9 | 0.7 | -0.27 | 2.7% | 12 | 0.88 | -0.073 |
| FinsH5^b^ | 1.6% | 17 | 0.87 | 0.25*** | 0.129 ± 0.015 | - | - | - | - | - | 0.5% | 5 | 0.3 | -0 | 2.7% | 10 | 0.83 | 0.031 |
| FinsI12^b^ | - | - | - | - | - | 1.7% | 5 | 0.68 | -0.04 | - | 1.1% | 3 | 0.1 | -0.05 | 0.0% | 3 | 0.28 | -0.163 |
| Frub29^c^ | 0.0% | 14 | 0.76 | 0.089* | - | - | - | - | - | - | 1.1% | 6 | 0.7 | 0 | - | - | - | - |
| Frub38^c^ | 0.0% | 10 | 0.81 | -0.03 | - | 3.4% | 4 | 0.58 | 0.28* | - | 4.7% | 5 | 0.7 | -0.01 | 0.0% | 3 | 0.64 | -0.235 |
| **Total** |  | 109 |  |  |  |  | 76 |  |  |  |  | 68 |  |  |  | 56 |  |  |
| **Mean** |  | 10.9 | 0.79 | 0.054 |  |  | 8.4 | 0.69 | 0.147 |  |  | 7.56 | 0.58 | -0.021 |  | 7.0 | 0.64 | -0.056 |

References: ^a^ Heer et al. 2012, ^b^ Vignes et al. 2006, ^c^ Crozier et al. 2007

**References**

Crozier YC, Jia XC, Yao JY, Field AR, Cook JM, et al. (2007) Microsatellite primers for *Ficus racemosa* and *Ficus rubiginosa*. Mol Ecol Notes 7: 57-59.

Heer K, Machado CA, Himler AG, Herre EA, Kalko EKV, et al. (2012) Anonymous and EST-based Microsatellite DNA markers that transfer broadly across the fig tree genus (*Ficus* L. Moraceae). Am J Bot 99: e330-e333

Vignes H, Hossaert-Mckey M, Beaune D, Fevre D, Anstett MC, et al. (2006) Development and characterization of microsatellite markers for a monoecious *Ficus* species, *Ficus insipida*, and cross-species amplification among different sections of *Ficus*. Mol Ecol Notes 6: 792-795
