# Supplementary material for: Spatial Scales of Genetic Structure in Free-Standing and Strangler Figs (Ficus, Moraceae) Inhabiting Neotropical Forests
Source: PLoS One. 2015 Jul 30;10(7):e0133581. doi: 10.1371/journal.pone.0133581 (PMC4520606; doi:10.1371/journal.pone.0133581)
Supplement: S2 Table — For each marker, we provide the percentage of missing data, the number of alleles (Na), the expected heterozygosity (He) and the inbreeding coefficient (FIS). (DOCX) [file pone.0133581.s011.docx]

**S2 Table**: Sampling sites of *F. inspida* in Panama, Costa Rica and Peru with the number of samples per plot (n) and the spatial coordinates of the locality.

| Country | Sampling plot | Region | n | Coordinates |
| --- | --- | --- | --- | --- |
| Panama | Barro Colorado Island | Panamá | 190 | 9°09’N, 79°51’W |
| Panama | Pipeline Road, P.N. Soberanía | Panamá | 155 | 9°08’N, 79°43’W |
| Panama | Eastern sector of P.N. Soberanía | Panamá | 28 | 9°05’N, 79°39’W |
| Panama | Forest close to the town of Gamboa | Panamá | 30 | 9°07’N, 79°42’W |
| Panama | P.N. San Lorenzo | Colón | 23 | 9°18’N, 79°57’W |
| Costa Rica | Tirimbina Biological Reserve | Sarapiquí | 27 | 10°24’N, 84°06’W |
| Peru | Highway between Iquitos and Nauta | Loreto | 5 | 4°02’N, 73°26’W |
| Peru | Rìo Amazonas east of Iquitos | Loreto | 7 | 3°32’N, 73°07’W |
| Peru | Río Ucayali east of Jenaro Herrera | Loreto | 5 | 4°54’N, 73°41’W |
| Peru | Yurimaguas | Loreto | 4 | 5°53’N, 76°11’W |
| Peru | Cainarachi | San Martín | 3 | 6°18’N, 76°16’W |
| Peru | Tarapoto | San Martín | 3 | 6°27’N, 76°19’W |
